# 1-Eth­oxy­methyl-5-methyl-9-phenyl-6,7,8,9-tetra­hydro-1*H*-pyrimido[4,5-*b*][1,4]diazepine-2,4(3*H*,5*H*)-dione

**DOI:** 10.1107/S1600536812014985

**Published:** 2012-04-18

**Authors:** Gong Li, Xiaowei Wang, Zhili Zhang, Junyi Liu

**Affiliations:** a Department of Chemical Biology, School of Pharmaceutical Sciences, Peking University, Beijing 100191, People’s Republic of China

## Abstract

The title compound, C_17_H_22_N_4_O_3_, comprises a 1,4-diazepine ring in a twist-boat conformation fused to a pyrimidine ring. The dihedral angle between the pyrimidine and phenyl rings is 80.8 (1)°. The crystal packing features N—H⋯O and C—H⋯O hydrogen bonds.

## Related literature

For the preparation of 2,4-dimeth­oxy-5-methyl-9-phenyl-8,9-dihydro-5*H*- pyrimido[4,5-*b*][1,4]diazepin-6(7*H*), see: Li *et al.* (2012 ▶). For the biological activity of compounds with a pyrimidodiazepine scaffold, see: Ferreira *et al.* (2009 ▶); Gracias *et al.* (2008 ▶); Insuasty *et al.* (2008 ▶); Chen *et al.* (2012 ▶). The title compound was obtained during work on the structural modification of our previously reported HIV-1 reverse transcriptase inhibitor, see: Wang *et al.* (2006 ▶).
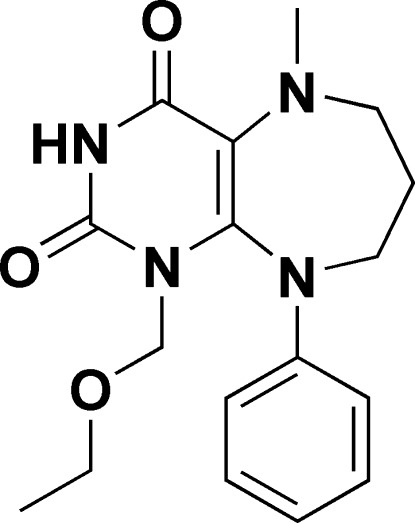


## Experimental

### 

#### Crystal data

C_17_H_22_N_4_O_3_
*M* *_r_* = 330.39
Monoclinic, 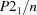

*a* = 13.831 (3) Å
*b* = 8.9904 (18) Å
*c* = 14.978 (3) Å
β = 112.79 (3)°
*V* = 1717.1 (6) Å^3^
*Z* = 4
Mo *K*α radiation
μ = 0.09 mm^−1^
*T* = 298 K
0.40 × 0.30 × 0.30 mm

#### Data collection

Rigaku R-AXIS RAPID IPdiffractometer
Absorption correction: multi-scan (*ABSCOR*; Higashi, 1995 ▶) *T*_min_ = 0.965, *T*_max_ = 0.974
7151 measured reflections
3921 independent reflections
1601 reflections with *I* > 2σ(*I*)
*R*_int_ = 0.041

#### Refinement

*R*[*F* ^2^ > 2σ(*F* ^2^)] = 0.079
*wR*(*F* ^2^) = 0.250
*S* = 0.86
3921 reflections
218 parameters
H atoms treated by a mixture of independent and constrained refinement
Δρ_max_ = 0.70 e Å^−3^
Δρ_min_ = −0.59 e Å^−3^

### 

Data collection: *RAPID-AUTO* (Rigaku, 2000 ▶); cell refinement: *RAPID-AUTO*; data reduction: *CrystalStructure* (Rigaku/MSC, 2000 ▶); program(s) used to solve structure: *SHELXS97* (Sheldrick, 2008 ▶); program(s) used to refine structure: *SHELXL97* (Sheldrick, 2008 ▶); molecular graphics: *SHELXTL* (Sheldrick, 2008 ▶); software used to prepare material for publication: *SHELXL97*.

## Supplementary Material

Crystal structure: contains datablock(s) I, global. DOI: 10.1107/S1600536812014985/kp2397sup1.cif

Structure factors: contains datablock(s) I. DOI: 10.1107/S1600536812014985/kp2397Isup2.hkl

Supplementary material file. DOI: 10.1107/S1600536812014985/kp2397Isup3.cdx

Supplementary material file. DOI: 10.1107/S1600536812014985/kp2397Isup4.cdx

Supplementary material file. DOI: 10.1107/S1600536812014985/kp2397Isup5.cml

Additional supplementary materials:  crystallographic information; 3D view; checkCIF report

## Figures and Tables

**Table 1:** Hydrogen-bond geometry (Å, °)

*D*—H⋯*A*	*D*—H	H⋯*A*	*D*⋯*A*	*D*—H⋯*A*
N1—H1*A*⋯O2^i^	0.95 (4)	1.91 (4)	2.862 (4)	175 (3)
C13—H13⋯O1^ii^	0.93	2.49	3.397 (5)	164

Symmetry codes: (i) 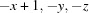; (ii) 
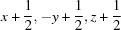
.

**Fig. 1.**
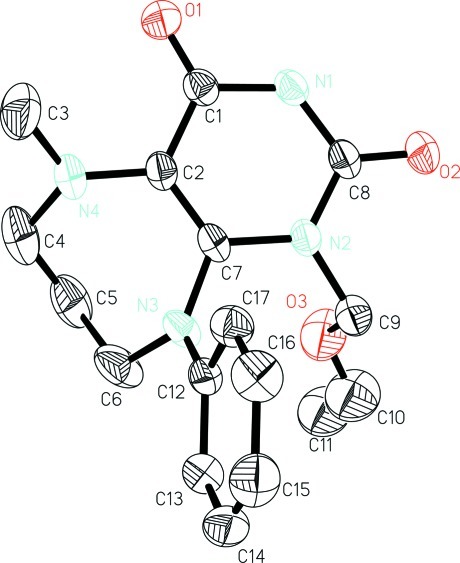
A view of compound (I), showing the atom-labelling scheme. The non-H atoms are shown with displacement ellipsoids drawn at the 50% probability level. H atoms are represented by circles of arbitrary size.

**Fig. 2.**
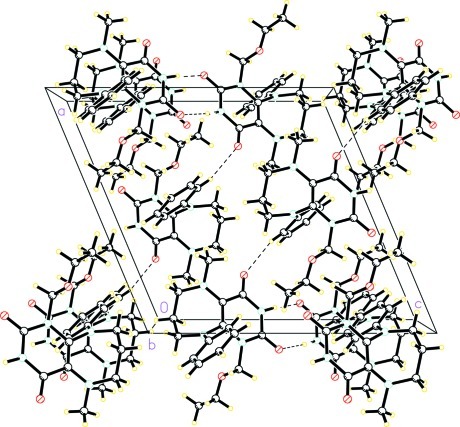
The packing of (I), viewed down the b axis. The intermolecular hydrogen bonds are denoted by dashed lines.

## supplementary crystallographic information

### Comment

The title compound (Fig. 1) belongs to a class having pyrimidodiazepine scaffold which in recent years has exhibited a range of biological activities such as antitumor agents (Insuasty *et al.*, 2008; Chen *et al.*, 2012), HIV-integrase inhibitors (Ferreira *et al.*, 2009) and receptor tyrosine kinase inhibitors (Gracias *et al.*, 2008). The title compound is gained during the structural modification work of our previously reported HIV-1 reverse transcriptase inhibitor (Wang *et al.*, 2006). The main goal of this modification is to enhance the physicochemical properties and the flexibility of the seven-membered ring fused to the pyrimidine ring.

In the title compound, the dihedral angle between the phenyl ring and the pyrimidine ring is 80.8 (1)°. The diazepine ring exhibits a twist-boat conformation. To make a clear description of this boat conformation, the C4, C6, N3 and N4 atoms are regarded as coplanar, so that the C2, C7 and C5 atoms lie at the same side of the plane.

In the crystal structure, the molecules are linked by an intermolecular hydrogen bond [N1-H···O 2.862 (4) and C13-H···O1 3.397 (5)Å] (Table 1, Fig. 2).

### Experimental

The important intermediate 2,4-dimethoxy-5-methyl-9-phenyl-8,9-dihydro-5*H*- pyrimido[4,5-*b*][1,4]diazepin-6(7*H*)-one was prepared according to our procedure (Li *et al.*, 2012). To a suspension of LiAlH_4_ (13 mg, 0.34 mmol, 2 equiv) in dry Et_2_O (15 mL) the above-mentioned intermediate lactam (0.17 mmol) was slowly added. Then the mixture was refluxed for 15 min, monitored by TLC. The mixture was cooled to rt, and quenched with a minimum amount of saturated aqueous sodium sulfate solution. Then the mixture was diluted with EtOAc, washed with brine. The organic layer was dried over anhydrous Na_2_SO_4_ and concentrated *in vacuo* to give a yellow oil. This was purified by column chromatograpy on silica gel. Elution with solvent mixture petroleum ether: EtOAc = 7: 1 gave the 2,4-dimethoxy-5-methyl-9-phenyl-6,7,8,9-tetrahydro-5*H*-pyrimido[4,5-*b*][1,4]diazepine in 76% yield. The reduction product (70 mg, 0.23 mmol) was dissolved in THF (25 mL) at room temperature and then concentrated hydrochloric acid (0.5 mL) was added. The mixture was refluxed for 8 h and monitored by TLC. Then the mixture was neutralised by saturated sodium bicarbonate solution and extracted twice with EtOAc (20 mL). The combined organic fractions were washed with brine, dried over anhydrous Na_2_SO4 and concentrated. Then recrystallization from MeOH/EtOAc/petroleum ether gave the colourless crystal product 43 mg, in 73% yield. To a suspension of the corresponding uracil (0.1 mmol) in chloroform (2 mL) *N*,*O*-bis(trimethylsilyl)acetamide (BSA) (0.25 mmol, 2.5equiv) was added and the stirring was continued until a clear solution was observed. Then chloromethyl ethyl ether (0.13 mmol) was added and the reaction mixture was stirred until no change in amount of the starting material. The reaction was quenched with a saturated solution of NaHCO_3_ and extracted twice with CHCl_3_ (10 mL). The combined organic fractions were washed with brine, dried over anhydrous Na_2_SO_4_ and concentrated *in vacuo* to give a yellow oil. This was purified by a thin layer chromatograpy on silica gel. Developing with dichloromethane: MeOH = 50: 1 gave the pure title compound in 69% yield. Then recrystallization from EtOAc/petroleum ether gave the colourless crystal. ^1^H NMR (400 MHz, CDCl_3_) δ 8.72 (s, 1H), 7.31 (t, *J* = 7.6 Hz,2*H*), 6.96 (t, *J* = 7.6 Hz, 1H), 6.85 (d, *J* = 7.6 Hz, 2H), 4.93 (s, 2H),3.77 (br s, 2H), 3.47 (q, *J* = 7.2 Hz, 2H), 3.01 (t, *J* = 5.6 Hz, 2H), 2.81(s, 3H), 1.89 (t, *J* = 5.6 Hz, 2H),1.11 (t, *J* = 7.2 Hz, 3H). ^13^C NMR (100 MHz, CDCl_3_) δ 162.14, 151.14, 145.05, 129.81, 121.32, 116.09, 72.96, 64.80, 51.25, 48.93,39.79, 23.01, 15.03. Anal. Calcd. for C_17_H_22_N_4_O_3_: C, 61.80; H, 6.71; N, 16. 96. Found: C, 61.71; H, 6.616; N, 17.00.

### Refinement

H atoms were positioned geometrically, with C—H = 0.93 Å for aryl, 0.97 Å for the methylene, and 0.96 Å for the methyl H atoms, N—H = 0.93 Å. U_iso_(H) = 1.5U_eq_(C) for the methyl groups, 1.2U_eq_(C) for methylene, and 0.07U_eq_(N).

### Crystal data

**Table:**

C_17_H_22_N_4_O_3_	*F*(000) = 704
*M**_r_* = 330.39	*D*_x_ = 1.278 Mg m^−^^3^
Monoclinic, *P*2_1_/*n*	Mo *K*α radiation, λ = 0.71073 Å
*a* = 13.831 (3) Å	θ = 2.6–27.5°
*b* = 8.9904 (18) Å	µ = 0.09 mm^−^^1^
*c* = 14.978 (3) Å	*T* = 298 K
β = 112.79 (3)°	Block, colourless
*V* = 1717.1 (6) Å^3^	0.40 × 0.30 × 0.30 mm
*Z* = 4	

### Data collection

**Table:**

Rigaku R-AXIS RAPID IP diffractometer	3921 independent reflections
Radiation source: fine-focus sealed tube	1601 reflections with *I* > 2σ(*I*)
Graphite monochromator	*R*_int_ = 0.041
Detector resolution: 10.00 pixels mm^-1^	θ_max_ = 27.5°, θ_min_ = 2.6°
Ω scans	*h* = −17→17
Absorption correction: multi-scan (*ABSCOR*; Higashi, 1995)	*k* = −11→11
*T*_min_ = 0.965, *T*_max_ = 0.974	*l* = −19→19
7151 measured reflections	

### Refinement

**Table:**

Refinement on *F*^2^	Secondary atom site location: difference Fourier map
Least-squares matrix: full	Hydrogen site location: inferred from neighbouring sites
*R*[*F*^2^ > 2σ(*F*^2^)] = 0.079	H atoms treated by a mixture of independent and constrained refinement
*wR*(*F*^2^) = 0.250	*w* = 1/[σ^2^(*F*_o_^2^) + (0.151*P*)^2^] where *P* = (*F*_o_^2^ + 2*F*_c_^2^)/3
*S* = 0.86	(Δ/σ)_max_ = 0.004
3921 reflections	Δρ_max_ = 0.70 e Å^−^^3^
218 parameters	Δρ_min_ = −0.59 e Å^−^^3^
0 restraints	Extinction correction: *SHELXL97* (Sheldrick, 2008), Fc^*^=kFc[1+0.001xFc^2^λ^3^/sin(2θ)]^-1/4^
Primary atom site location: structure-invariant direct methods	Extinction coefficient: 0.062 (7)

### Special details

**Table:**

Geometry. All e.s.d.'s (except the e.s.d. in the dihedral angle between two l.s. planes) are estimated using the full covariance matrix. The cell e.s.d.'s are taken into account individually in the estimation of e.s.d.'s in distances, angles and torsion angles; correlations between e.s.d.'s in cell parameters are only used when they are defined by crystal symmetry. An approximate (isotropic) treatment of cell e.s.d.'s is used for estimating e.s.d.'s involving l.s. planes.
Refinement. Refinement of *F*^2^ against ALL reflections. The weighted *R*-factor *wR* and goodness of fit *S* are based on *F*^2^, conventional *R*-factors *R* are based on *F*, with *F* set to zero for negative *F*^2^. The threshold expression of *F*^2^ > σ(*F*^2^) is used only for calculating *R*-factors(gt) *etc*. and is not relevant to the choice of reflections for refinement. *R*-factors based on *F*^2^ are statistically about twice as large as those based on *F*, and *R*– factors based on ALL data will be even larger.

### Fractional atomic coordinates and isotropic or equivalent isotropic displacement parameters (Å^2^)

**Table:**

	*x*	*y*	*z*	*U*_iso_*/*U*_eq_	
C1	0.3598 (3)	0.0927 (4)	0.1226 (2)	0.0529 (8)	
C2	0.3869 (3)	0.1940 (4)	0.2044 (2)	0.0509 (8)	
C3	0.2088 (3)	0.2532 (6)	0.1902 (3)	0.1021 (16)	
H3A	0.2026	0.2844	0.1269	0.153*	
H3B	0.1855	0.3317	0.2205	0.153*	
H3C	0.1665	0.1664	0.1847	0.153*	
C4	0.3437 (4)	0.1949 (5)	0.3481 (3)	0.0979 (15)	
H4A	0.3072	0.1067	0.3558	0.118*	
H4B	0.3181	0.2784	0.3738	0.118*	
C5	0.4578 (4)	0.1761 (5)	0.4074 (3)	0.0916 (15)	
H5A	0.4665	0.1515	0.4731	0.110*	
H5B	0.4834	0.0925	0.3820	0.110*	
C6	0.5239 (4)	0.3082 (5)	0.4105 (3)	0.0927 (15)	
H6A	0.5043	0.3886	0.4433	0.111*	
H6B	0.5966	0.2836	0.4485	0.111*	
C7	0.4813 (3)	0.2614 (3)	0.2368 (2)	0.0481 (8)	
C8	0.5306 (3)	0.1445 (4)	0.1140 (2)	0.0540 (9)	
C9	0.6648 (3)	0.2706 (4)	0.2460 (2)	0.0624 (10)	
H9A	0.7025	0.2656	0.2034	0.075*	
H9B	0.6723	0.3699	0.2732	0.075*	
C10	0.7968 (4)	0.1714 (8)	0.3765 (4)	0.1291 (15)	
H10A	0.8076	0.2688	0.4067	0.155*	
H10B	0.8390	0.1673	0.3377	0.155*	
C11	0.8383 (4)	0.0601 (7)	0.4539 (4)	0.1291 (15)	
H11A	0.7953	0.0577	0.4911	0.194*	
H11B	0.9088	0.0860	0.4954	0.194*	
H11C	0.8379	−0.0360	0.4259	0.194*	
C12	0.5293 (2)	0.5115 (4)	0.3007 (2)	0.0483 (8)	
C13	0.5958 (3)	0.5994 (4)	0.3752 (3)	0.0632 (10)	
H13	0.6339	0.5586	0.4358	0.076*	
C14	0.6045 (3)	0.7501 (4)	0.3579 (3)	0.0729 (11)	
H14	0.6477	0.8101	0.4081	0.087*	
C15	0.5513 (3)	0.8117 (4)	0.2692 (3)	0.0785 (12)	
H15	0.5590	0.9121	0.2586	0.094*	
C16	0.4866 (3)	0.7244 (4)	0.1964 (3)	0.0732 (11)	
H16	0.4497	0.7662	0.1359	0.088*	
C17	0.4749 (3)	0.5758 (4)	0.2106 (2)	0.0590 (9)	
H17	0.4305	0.5179	0.1598	0.071*	
H1A	0.420 (3)	0.018 (4)	0.027 (3)	0.070 (10)*	
N1	0.4323 (2)	0.0844 (3)	0.07953 (19)	0.0533 (7)	
N2	0.55515 (19)	0.2347 (3)	0.19371 (18)	0.0505 (7)	
N3	0.5151 (2)	0.3599 (3)	0.31555 (17)	0.0561 (8)	
N4	0.3169 (2)	0.2189 (4)	0.2482 (2)	0.0805 (10)	
O1	0.2790 (2)	0.0204 (3)	0.08886 (18)	0.0761 (8)	
O2	0.59235 (19)	0.1200 (3)	0.07421 (17)	0.0757 (8)	
O3	0.7038 (2)	0.1625 (4)	0.3210 (2)	0.1100 (12)	

### Atomic displacement parameters (Å^2^)

**Table:**

	*U*^11^	*U*^22^	*U*^33^	*U*^12^	*U*^13^	*U*^23^
C1	0.067 (2)	0.046 (2)	0.0511 (18)	−0.0031 (17)	0.0282 (17)	−0.0018 (16)
C2	0.061 (2)	0.0484 (19)	0.0545 (18)	−0.0016 (16)	0.0342 (16)	−0.0008 (15)
C3	0.078 (3)	0.119 (4)	0.127 (4)	0.010 (3)	0.060 (3)	−0.012 (3)
C4	0.135 (4)	0.098 (4)	0.098 (3)	0.014 (3)	0.085 (3)	0.013 (3)
C5	0.143 (4)	0.081 (3)	0.076 (3)	0.009 (3)	0.070 (3)	0.016 (2)
C6	0.152 (4)	0.084 (3)	0.053 (2)	−0.028 (3)	0.052 (3)	−0.007 (2)
C7	0.067 (2)	0.0424 (18)	0.0439 (16)	0.0054 (16)	0.0318 (16)	0.0012 (14)
C8	0.069 (2)	0.052 (2)	0.0523 (18)	0.0014 (17)	0.0350 (17)	−0.0020 (17)
C9	0.063 (2)	0.070 (3)	0.057 (2)	0.0059 (19)	0.0268 (17)	0.0037 (19)
C10	0.106 (3)	0.161 (4)	0.096 (3)	0.004 (3)	0.013 (2)	0.026 (3)
C11	0.106 (3)	0.161 (4)	0.096 (3)	0.004 (3)	0.013 (2)	0.026 (3)
C12	0.0493 (18)	0.051 (2)	0.0492 (18)	0.0033 (15)	0.0242 (15)	−0.0057 (16)
C13	0.066 (2)	0.066 (3)	0.060 (2)	−0.0027 (19)	0.0265 (18)	−0.0080 (19)
C14	0.076 (3)	0.060 (3)	0.089 (3)	−0.014 (2)	0.039 (2)	−0.024 (2)
C15	0.094 (3)	0.048 (2)	0.105 (3)	−0.002 (2)	0.051 (3)	0.001 (2)
C16	0.085 (3)	0.058 (3)	0.076 (3)	0.009 (2)	0.031 (2)	0.008 (2)
C17	0.062 (2)	0.054 (2)	0.061 (2)	0.0036 (17)	0.0227 (18)	−0.0037 (18)
N1	0.0655 (19)	0.0505 (17)	0.0509 (16)	−0.0029 (14)	0.0304 (14)	−0.0101 (14)
N2	0.0560 (17)	0.0541 (17)	0.0502 (15)	−0.0006 (13)	0.0303 (13)	−0.0030 (13)
N3	0.082 (2)	0.0504 (18)	0.0420 (14)	−0.0028 (14)	0.0310 (14)	−0.0031 (13)
N4	0.075 (2)	0.114 (3)	0.070 (2)	−0.0053 (19)	0.0478 (18)	−0.0150 (19)
O1	0.0730 (17)	0.0815 (19)	0.0795 (17)	−0.0219 (15)	0.0360 (14)	−0.0220 (15)
O2	0.0804 (18)	0.088 (2)	0.0769 (17)	−0.0075 (14)	0.0507 (15)	−0.0232 (14)
O3	0.073 (2)	0.168 (3)	0.0755 (19)	0.009 (2)	0.0146 (16)	0.035 (2)

### Geometric parameters (Å, º)

**Table:**

C1—O1	1.221 (4)	C9—O3	1.424 (4)
C1—N1	1.389 (4)	C9—N2	1.448 (4)
C1—C2	1.454 (4)	C9—H9A	0.9700
C2—C7	1.347 (4)	C9—H9B	0.9700
C2—N4	1.382 (4)	C10—O3	1.235 (5)
C3—N4	1.442 (5)	C10—C11	1.470 (7)
C3—H3A	0.9600	C10—H10A	0.9700
C3—H3B	0.9600	C10—H10B	0.9700
C3—H3C	0.9600	C11—H11A	0.9600
C4—N4	1.412 (5)	C11—H11B	0.9600
C4—C5	1.491 (6)	C11—H11C	0.9600
C4—H4A	0.9700	C12—C13	1.386 (4)
C4—H4B	0.9700	C12—C17	1.392 (4)
C5—C6	1.488 (6)	C12—N3	1.407 (4)
C5—H5A	0.9700	C13—C14	1.393 (5)
C5—H5B	0.9700	C13—H13	0.9300
C6—N3	1.456 (4)	C14—C15	1.362 (5)
C6—H6A	0.9700	C14—H14	0.9300
C6—H6B	0.9700	C15—C16	1.360 (5)
C7—N3	1.403 (4)	C15—H15	0.9300
C7—N2	1.424 (4)	C16—C17	1.372 (5)
C8—O2	1.235 (4)	C16—H16	0.9300
C8—N1	1.366 (4)	C17—H17	0.9300
C8—N2	1.373 (4)	N1—H1A	0.95 (4)
			
O1—C1—N1	119.3 (3)	O3—C10—H10A	107.7
O1—C1—C2	125.4 (3)	C11—C10—H10A	107.7
N1—C1—C2	115.3 (3)	O3—C10—H10B	107.7
C7—C2—N4	121.0 (3)	C11—C10—H10B	107.7
C7—C2—C1	118.9 (3)	H10A—C10—H10B	107.1
N4—C2—C1	120.1 (3)	C10—C11—H11A	109.5
N4—C3—H3A	109.5	C10—C11—H11B	109.5
N4—C3—H3B	109.5	H11A—C11—H11B	109.5
H3A—C3—H3B	109.5	C10—C11—H11C	109.5
N4—C3—H3C	109.5	H11A—C11—H11C	109.5
H3A—C3—H3C	109.5	H11B—C11—H11C	109.5
H3B—C3—H3C	109.5	C13—C12—C17	119.0 (3)
N4—C4—C5	115.5 (3)	C13—C12—N3	121.0 (3)
N4—C4—H4A	108.4	C17—C12—N3	120.0 (3)
C5—C4—H4A	108.4	C12—C13—C14	118.9 (3)
N4—C4—H4B	108.4	C12—C13—H13	120.6
C5—C4—H4B	108.4	C14—C13—H13	120.6
H4A—C4—H4B	107.5	C15—C14—C13	121.6 (4)
C6—C5—C4	115.1 (4)	C15—C14—H14	119.2
C6—C5—H5A	108.5	C13—C14—H14	119.2
C4—C5—H5A	108.5	C16—C15—C14	119.1 (4)
C6—C5—H5B	108.5	C16—C15—H15	120.4
C4—C5—H5B	108.5	C14—C15—H15	120.4
H5A—C5—H5B	107.5	C15—C16—C17	121.2 (4)
N3—C6—C5	114.0 (3)	C15—C16—H16	119.4
N3—C6—H6A	108.8	C17—C16—H16	119.4
C5—C6—H6A	108.8	C16—C17—C12	120.2 (3)
N3—C6—H6B	108.8	C16—C17—H17	119.9
C5—C6—H6B	108.8	C12—C17—H17	119.9
H6A—C6—H6B	107.7	C8—N1—C1	126.6 (3)
C2—C7—N3	123.2 (3)	C8—N1—H1A	113 (2)
C2—C7—N2	121.8 (3)	C1—N1—H1A	119 (2)
N3—C7—N2	115.0 (3)	C8—N2—C7	120.9 (3)
O2—C8—N1	121.3 (3)	C8—N2—C9	117.1 (3)
O2—C8—N2	122.8 (3)	C7—N2—C9	120.2 (3)
N1—C8—N2	115.9 (3)	C7—N3—C12	120.0 (2)
O3—C9—N2	105.8 (3)	C7—N3—C6	119.6 (3)
O3—C9—H9A	110.6	C12—N3—C6	120.0 (3)
N2—C9—H9A	110.6	C2—N4—C4	122.3 (3)
O3—C9—H9B	110.6	C2—N4—C3	120.2 (3)
N2—C9—H9B	110.6	C4—N4—C3	117.1 (3)
H9A—C9—H9B	108.7	C10—O3—C9	117.7 (4)
O3—C10—C11	118.3 (5)		
			
O1—C1—C2—C7	176.9 (3)	N1—C8—N2—C9	165.3 (3)
N1—C1—C2—C7	−5.3 (4)	C2—C7—N2—C8	3.3 (5)
O1—C1—C2—N4	−2.4 (5)	N3—C7—N2—C8	−177.8 (3)
N1—C1—C2—N4	175.4 (3)	C2—C7—N2—C9	−161.2 (3)
N4—C4—C5—C6	63.4 (5)	N3—C7—N2—C9	17.8 (4)
C4—C5—C6—N3	−56.3 (5)	O3—C9—N2—C8	−93.3 (3)
N4—C2—C7—N3	−0.1 (5)	O3—C9—N2—C7	71.8 (4)
C1—C2—C7—N3	−179.5 (3)	C2—C7—N3—C12	−112.7 (4)
N4—C2—C7—N2	178.7 (3)	N2—C7—N3—C12	68.4 (4)
C1—C2—C7—N2	−0.6 (5)	C2—C7—N3—C6	60.4 (5)
C17—C12—C13—C14	1.1 (5)	N2—C7—N3—C6	−118.5 (4)
N3—C12—C13—C14	−177.8 (3)	C13—C12—N3—C7	−156.5 (3)
C12—C13—C14—C15	−1.5 (5)	C17—C12—N3—C7	24.5 (4)
C13—C14—C15—C16	1.2 (6)	C13—C12—N3—C6	30.4 (5)
C14—C15—C16—C17	−0.5 (6)	C17—C12—N3—C6	−148.6 (4)
C15—C16—C17—C12	0.2 (5)	C5—C6—N3—C7	−23.0 (6)
C13—C12—C17—C16	−0.5 (5)	C5—C6—N3—C12	150.2 (4)
N3—C12—C17—C16	178.4 (3)	C7—C2—N4—C4	−55.7 (5)
O2—C8—N1—C1	173.7 (3)	C1—C2—N4—C4	123.6 (4)
N2—C8—N1—C1	−7.3 (5)	C7—C2—N4—C3	132.0 (4)
O1—C1—N1—C8	−172.3 (3)	C1—C2—N4—C3	−48.7 (5)
C2—C1—N1—C8	9.8 (5)	C5—C4—N4—C2	13.9 (6)
O2—C8—N2—C7	179.3 (3)	C5—C4—N4—C3	−173.6 (4)
N1—C8—N2—C7	0.4 (4)	C11—C10—O3—C9	−179.7 (5)
O2—C8—N2—C9	−15.7 (5)	N2—C9—O3—C10	−179.3 (4)

### Hydrogen-bond geometry (Å, º)

**Table:**

*D*—H···*A*	*D*—H	H···*A*	*D*···*A*	*D*—H···*A*
N1—H1*A*···O2^i^	0.95 (4)	1.91 (4)	2.862 (4)	175 (3)
C13—H13···O1^ii^	0.93	2.49	3.397 (5)	164

Symmetry codes: (i) −*x*+1, −*y*, −*z*; (ii) *x*+1/2, −*y*+1/2, *z*+1/2.
